# Maternal Exercise and Offspring Cardiac Regenerative Potential: Roles of Apelin and α-Ketoglutarate

**DOI:** 10.3390/biology15161422

**Published:** 2026-08-18

**Authors:** Guanfeng Qin, Fan Li, Haiwang Shi

**Affiliations:** 1 Laboratory of Regenerative Medicine in Sports Science, School of Physical Education and Sports Science, South China Normal University, Guangzhou 510006, China; 2023020981@m.scnu.edu.cn; 2 Department of Cardiovascular Medicine, Physiology and Biomedical Engineering, Center for Regenerative Biotherapeutics, Mayo Clinic Arizona, Scottsdale, AZ 85259, USA

**Keywords:** cardiomyocyte proliferation, maternal exercise, epigenetics, cardiovascular disease

## Abstract

Cardiovascular disease causes the largest number of deaths across the world. The weak regenerative ability of adult hearts originates from limited cell proliferation and polyploidization of postnatal cardiomyocytes, which severely hinders tissue repair after myocardial injury. Fetal development represents a critical window during which maternal physiological and metabolic status can shape long-term offspring cardiac structure and function. Maternal exercise represents a safe nonpharmacological prenatal intervention that confers long-term benefits to offspring health. Available evidence indicates that maternal exercise enhances placental apelin secretion and elevates α-ketoglutarate (α-KG) levels in fetal brown adipose tissue, liver, and skeletal muscle. The apelin and α-KG signaling cascade participates in epigenetic regulation and confers protective effects on offspring metabolic health. Independent animal experiments have further validated that supplementation with either apelin or α-KG alleviates myocardial infarction (MI)-induced cardiac injury in adult mice, with α-KG showing direct evidence of reactivating cardiomyocyte proliferation. Nevertheless, it remains poorly elucidated whether maternal exercise could modulate fetal cardiomyocyte proliferative capacity in offspring through apelin- or α-KG-associated signaling. This narrative review integrates current evidence and proposes a testable framework in which maternal exercise influences fetal cardiac growth and regenerative potential through placental, metabolic, and epigenetic signaling. Because direct evidence remains limited, this model should be regarded as a hypothesis that requires further experimental validation.

## Simple Summary

Maternal exercise-linked metabolic programming may carry tentative implications for long-term cardiac health in offspring. Maternal exercise modulates apelin, SOD3, AMPK, and α-KG-related signaling cascades within the placenta and non-cardiac fetal tissues. Separately, α-KG directly participates in the regulation of cardiomyocyte cell cycle activity and cardiac repair. It remains unclear whether these signaling mechanisms function within the developing fetal heart. This review puts forward a hypothesis stating that maternal exercise shapes fetal cardiac development and regenerative capacity via placental metabolic and epigenetic signaling. Future investigations are required to clarify tissue-specific and causal functions of apelin and α-KG in fetal and neonatal cardiomyocyte proliferation. Further work should also test whether maternal exercise-linked developmental programming provides persistent protection against cardiac injury in adulthood.

## 1. Introduction

Cardiovascular disease (CVD) remains the leading cause of death globally. Recent findings from authoritative cardiovascular journals predict a substantial rise in CVD prevalence worldwide between 2025 and 2050, accompanied by a 73.4% increase in associated mortality [1]. Specifically, CVD-attributable deaths are forecasted to climb from 20.5 million in 2025 to 35.6 million by 2050 [1]. These statistics highlight an urgent demand for improved CVD prevention and therapeutic approaches, reinforcing the necessity to develop innovative, effective, and safe interventional strategies. The limited regenerative capacity of the heart is a critical biological bottleneck in advancing CVD therapeutics. During mammalian cardiac development, multipotent cardiac progenitor cells originating from the first and second heart fields differentiate into cardiomyocytes. These newly formed immature cardiomyocytes maintain robust proliferation throughout embryogenesis and drive cardiac growth and chamber morphogenesis [2]. Cardiac growth shifts predominantly toward cardiomyocyte hypertrophy rather than hyperplasia after birth [3]. Concurrently, cardiomyocyte proliferative activity declines markedly, and annual cardiomyocyte turnover in the adult human heart is estimated to be only approximately 0.3–1% [4]. This minimal regenerative reserve is insufficient to replenish large numbers of cardiomyocytes lost upon severe cardiac injury. Injured myocardial tissue is eventually substituted with fibrotic scar tissue, which initiates pathological ventricular remodeling, gradual cardiac dysfunction, and eventual heart failure [5].

**Figure 1:**
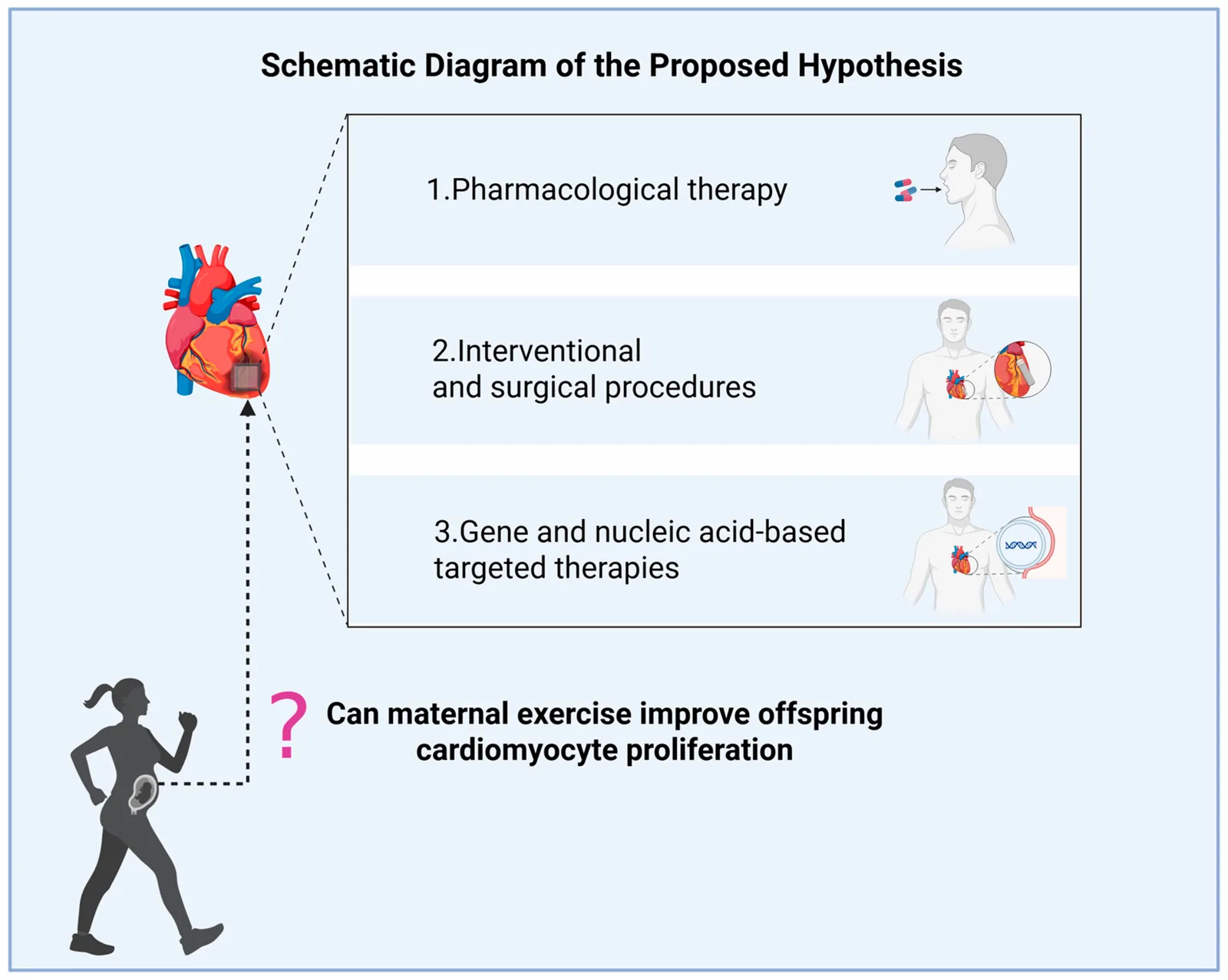
Schematic Diagram of the Proposed Hypothesis. Therapeutic interventions for CVD are divided into three major modalities: pharmacological therapy, interventional and surgical procedures, as well as gene and nucleic acid-based targeted therapies. The present review seeks to clarify whether maternal exercise improves offspring cardiomyocyte proliferation. Created with BioRender.com.

Current CVD therapeutic approaches mainly include pharmacological interventions [6,7], interventional and surgical treatments [8,9], as well as gene and nucleic acid-based targeted therapies [10,11,12]. Relevant progress has been achieved in single-gene disorder management, multi-gene risk evaluation, and delivery system optimization [13]. Nevertheless, these approaches face prominent translational limitations. Notably, insufficient attention has been paid to how developmental factors shape long-term adult cardiovascular health. As the earliest organ formed during embryogenesis [14], the mammalian heart develops through three successive stages, including early patterning, morphogenesis and maturation, with functional differentiation largely completed in the early postnatal period [15,16]. Extensive research has examined how regular physical activity during pregnancy influences offspring metabolic homeostasis, neural development and skeletal muscle function [17,18,19,20]. However, studies investigating whether maternal exercise modulates offspring cardiomyocyte proliferative potential remain limited. Notably, fetal cardiomyocyte proliferation determines the final cardiomyocyte pool formed before birth [2]. This prenatal cellular reserve shapes baseline adult cardiac function yet cannot be directly equated to cardiac regenerative capacity after myocardial injury [21,22]. As a promising prenatal intervention, maternal exercise may facilitate fetal cardiomyocyte proliferation to support favorable basal cardiac phenotypes in offspring. Such developmental programming may also offer valuable insights for strategies aimed at reactivating cardiomyocyte proliferation following adult myocardial infarction (MI) (Figure 1).

Existing animal data demonstrate that maternal exercise stimulates placental apelin secretion and elevates fetal α-ketoglutarate (α-KG) concentrations in brown adipose tissue, liver, and skeletal muscle and adipose tissue, mediating intergenerational metabolic programming [18,19,23,24]. Comparable evidence within cardiac tissue is still lacking. The fetal heart relies on glycolysis and epigenetic remodeling to sustain cardiomyocyte proliferation [15,25,26]. Apelin and α-KG function as two pivotal metabolic epigenetic regulators controlling this developmental program. To fill this research gap, we integrate available cross-tissue evidence, cardiac mechanistic data and intergenerational exercise studies to propose a tentative regulatory cascade linking maternal exercise to fetal cardiac apelin and α-KG-related signaling. This Perspective focuses on apelin and α-KG signaling in fetal myocardium. Relative to other exercise-responsive mediators such as superoxide dismutase (SOD) [18,19], this axis provides a unique metabolic and epigenetic framework to interpret the cardiac advantages conferred by prenatal physical activity.

We formulate the central hypothesis of this review as follows. Maternal exercise has the potential to boost cardiomyocyte proliferation in progeny, with the apelin/α-KG axis serving as a plausible candidate mediator rather than a fully validated signaling axis. Improved cardiomyocyte development during gestation strengthens baseline cardiac function in adult offspring and may further enhance cardiac regenerative potential after myocardial infarction. We acknowledge that enhanced fetal cardiomyocyte proliferation expands the postnatal cardiomyocyte reservoir, yet definitive evidence confirming that such developmental changes facilitate post-injury cardiac repair remains absent. Collectively, this signaling framework offers a novel intergenerational perspective for advancing CVD therapeutic research.

## 2. Regulatory Mechanisms of Cardiomyocyte Proliferation and Associations with Maternal Exercise

**Figure 2:**
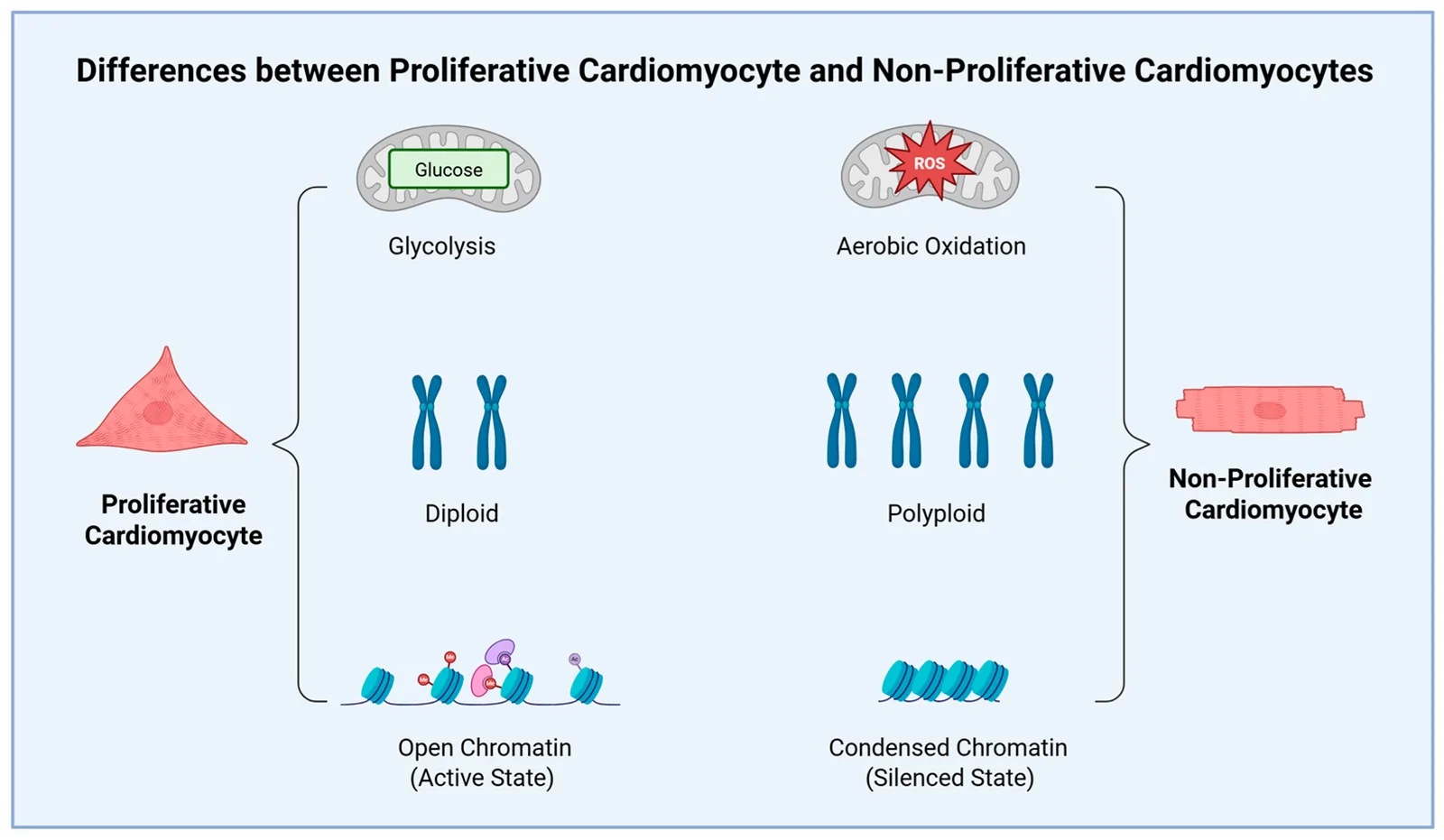
Differences between Proliferative and Non-Proliferative Cardiomyocytes. Alterations in metabolic pathways, chromosomal ploidy, and epigenetic regulation predominantly drive cell cycle withdrawal, which consequently results in the limited regenerative competence of adult cardiomyocytes. Created with BioRender.com.

The regenerative potential of the mammalian heart shifts drastically during early postnatal development. Fetal and neonatal cardiomyocytes possess intrinsic proliferative competence to sustain myocardial growth [27]. This regenerative capacity nevertheless fades rapidly alongside cardiomyocyte maturation after birth [28,29], including metabolic switching from glycolysis to mitochondrial oxidative phosphorylation and fatty acid oxidation [30], increased reactive oxygen species (ROS) production [31], binucleation and polyploidization [32,33], sarcomere maturation [34,35,36], and epigenetic repression of cell-cycle genes [37] (Figure 2). Consequently, the adult heart possesses only negligible capacity to replenish cardiomyocytes lost upon cardiac injury. This deficiency drives fibrotic scar formation and persistent cardiac functional deterioration [38].

Cardiomyocyte proliferative capacity is controlled by a complex network of intrinsic molecular pathways that regulate cell-cycle entry, DNA synthesis, mitosis, and cytokinesis. Core cell-cycle regulators, including cyclins [31,39], cyclin-dependent kinases (CDKs), and E2F transcription factor 2 E2 promoter-binding factor transcription factors, directly promote cardiomyocyte cell-cycle progression, whereas cell-cycle inhibitors such as P21, P27 restrict proliferation [40]. Beyond core cell-cycle machinery, developmental and transcriptional regulators also serve as essential modulators of this process. For example, GATA4 [41,42], TBX20 [43,44,45] and FOXM1 [46] have been implicated in promoting cardiomyocyte cell-cycle activity, whereas MEIS1 and HOXB13 contribute to postnatal cell-cycle arrest [47,48]. Several major signaling pathways further modulate these intrinsic cell-cycle programs, including Hippo-YAP [49], PI3K-Akt-mTOR [50], ERK [51], Notch [52,53,54], Wnt/β-catenin [55], and neuregulin/ERBB2 signaling [56]. Among these, Hippo-YAP signaling is particularly important because nuclear YAP promotes the expression of pro-proliferative genes [37,55], while postnatal Hippo pathway activation limits YAP activity and contributes to cardiomyocyte cell-cycle exit [57]. Similarly, PI3K-Akt-mTOR and ERK signaling can promote cell growth and proliferation, while neuregulin/ERBB2 signaling has been shown to stimulate cardiomyocyte cell-cycle re-entry and improve cardiac repair in experimental models [56]. These pathways collectively determine whether cardiomyocytes remain in a proliferative state or undergo permanent cell-cycle withdrawal.

In addition to these canonical signaling pathways, cardiomyocyte proliferation is strongly regulated by metabolic and epigenetic remodeling during postnatal maturation [15]. Before birth and during early neonatal life, cardiomyocytes rely more heavily on glycolysis, a metabolic state that is generally permissive for proliferation [58]. After birth, increased oxygen availability promotes a shift toward mitochondrial oxidative phosphorylation and fatty acid oxidation, leading to increased mitochondrial activity, ROS production, activation of the DNA damage response, and cell-cycle arrest [25]. Accompanying this metabolic remodeling is comprehensive structural and nuclear maturation, including sarcomere rearrangement, increased cellular stiffness, binucleation, and polyploidization. All of these phenotypic changes impair the capacity of cardiomyocytes to complete cytokinesis [32]. Epigenetic mechanisms further reinforce this non-proliferative state by repressing cell-cycle genes through DNA methylation and histone modifications, such as increased repressive H3K27me3 at promoters of proliferation-related genes [59].

Because cardiomyocyte proliferative capacity is highly sensitive to developmental, metabolic, and epigenetic cues, pregnancy may represent a critical window during which maternal physiological status shapes offspring cardiac growth and regenerative potential. Maternal exercise has been widely studied as a physiological intervention that improves offspring health [17,18,19,23,24,60,61]. Mechanistically, these beneficial effects converge on two key exercise-responsive placental mediators, apelin and α-KG [19,23,24]. Both molecules activate AMPK-dependent metabolic pathways to enhance mitochondrial function [19,23]. Accordingly, apelin and α-KG act as core effector molecules that may underpin the intergenerational metabolic health benefits of maternal exercise, though their cardiac-specific effects remain unconfirmed.

Collectively, these findings demonstrate that maternal exercise confers beneficial developmental cues to offspring via placental, endocrine, metabolic, and epigenetic pathways. Nevertheless, whether these analogous mechanisms govern cardiac development and the proliferative potential of cardiomyocytes in offspring remains largely unexplored.

## 3. Evidence That Maternal Exercise Influences Offspring Cardiac Development and Function

Several studies support the beneficial effects of maternal exercise on offspring cardiac development and function. In a maternal high-fat diet model, maternal exercise preserved cardiovascular health in female offspring by improving cardiomyocyte contractility, calcium handling, mitochondrial respiration, and RyR2 activity while reducing RyR2 oxidation [62]. In obese pregnancies, maternal exercise also prevented cardiac hypertrophy and systolic dysfunction in male offspring and increased the expression of cardiac calcium-handling proteins [63]. In fetal mouse hearts, maternal exercise enhanced mitochondrial biogenesis and dynamics by increasing the expression of NRF1, NRF2, and MFN2 [64]. In diabetic pregnancies, maternal exercise reduced fetal cardiac oxidative stress, restored myocardial cell proliferation, and lowered the incidence of congenital heart defects [65]. Human studies further showed that prenatal exercise lowered fetal heart rate, increased fetal heart-rate variability, and improved fetal right ventricular stroke volume and cardiac output [66,67]. These cardiac autonomic benefits may persist after birth, as infants of physically active mothers exhibited greater heart-rate variability at one month of age [68]. Collectively, these observations confirm maternal exercise confers protective effects on cardiac development, metabolism and function, yet direct evidence linking this intervention to enhanced offspring cardiac regeneration remains absent. Considering apelin and α-KG mediate the intergenerational metabolic and skeletal muscle benefits induced by maternal exercise, we hypothesize that these two factors could serve as functional mediators if gestational exercise modulates fetal cardiomyocyte regenerative capacity via cardiac-specific signaling in progeny.

This hypothesis builds upon the assumption that maternal exercise exerts measurable regulatory effects on offspring cardiomyocyte regenerative potential. Meanwhile, safe exercise thresholds for pregnant individuals remain incompletely defined. One prior study reported that elite female athletes performing strenuous activity exceeding 90% of maximal maternal heart rate between gestational weeks 23 and 29 presented fetal bradycardia and elevated umbilical artery pulsatility index. These changes coincided with a reduction in mean uterine artery blood flow to less than half of baseline values. Although fetal heart rate recovers after exercise cessation, these physiological shifts suggest that excessive gestational exercise may carry latent risks for fetal development [69]. Importantly, robust evidence linking maternal exercise to persistent adverse developmental outcomes in offspring is still lacking, and few studies systematically explore whether specific exercise regimens induce long-term detrimental effects. On the other hand, recent work supports that pregnant people and their fetuses can tolerate high-intensity resistance training, even when movements involve the Valsalva manoeuvre [70]. Given the risks associated with overexertion, pregnant individuals should follow standardized evidence-based exercise guidelines.

Current studies on maternal exercise also carry notable limitations. First, most animal interventions adopt aerobic exercise protocols such as treadmill training and voluntary wheel running [18,19,23,24,71], limiting the diversity of exercise modalities under investigation. How physiological stress induced by these dominant exercise paradigms shapes developmental outcomes in offspring remains insufficiently characterized. Second, quantitative dose benchmarks connecting maternal exercise to offspring health advantages have not been established. Further rigorous research is required to clarify the dose–response relationship underlying maternal exercise-driven developmental programming in progeny.

## 4. Potential Roles of Apelin and α-KG as Mediators of Maternal Exercise-Induced Cardiomyocyte Proliferation in Offspring

### 4.1. Apelin: An Exercise-Responsive Mediator of Cardiovascular and Metabolic Programming

Apelin is the endogenous ligand of the G protein-coupled receptor APJ and a vital modulator of cardiovascular homeostasis, systemic metabolism and tissue repair [72]. Exogenous apelin administration alleviates post-myocardial infarction (MI) cardiac fibrosis, scarring and pathological ventricular remodeling, conferring robust cardioprotective and pro-regenerative effects [73,74]. Apelin expression is hypoxia-inducible: hypoxia stabilizes HIF-1α to upregulate apelin, forming an oxygen-sensitive cascade that controls vascular adaptation [75]. Microparticle-delivered apelin further attenuates acute and chronic post-MI cardiac damage and adverse remodeling in mice [72]. However, it remains unconfirmed whether apelin directly drives cell cycle re-entry in fetal, neonatal, or adult cardiomyocytes.

PKM2-dependent glycolysis is tightly linked to cardiomyocyte proliferative capacity. After MI in adult mice, elevated PKM2 shifts cardiac metabolism toward glycolysis, boosts cardiomyocyte cycling activity, and improves long-term cardiac function and survival [25]. Animal studies confirm maternal exercise enhances placental apelin release and activates apelin signaling in fetal skeletal muscle and brown adipose tissue [18,23,24]. Apelin stabilizes fetal metabolic homeostasis and mitochondrial function via AMPK activation [18,23]. In vascular smooth muscle cells, apelin-13 triggers PKM2-mediated glycolytic reprogramming to facilitate cell cycle progression [76], a metabolic program likely conserved in cardiomyocytes. Collectively, these cross-tissue data support a tentative apelin–AMPK–PKM2 signaling cascade that may regulate fetal myocardial development following maternal exercise.

Notably, no in vivo work has quantified apelin abundance in the fetal myocardium of exercised dams, nor verified that cardiac apelin initiates PKM2-driven glycolysis to expand cardiomyocyte populations. This mechanistic model therefore awaits direct experimental validation in cardiac tissue.

### 4.2. α-KG: A Metabolic–Epigenetic Mediator of Cardiomyocyte Proliferative Potential

Cardiomyocytes rely on multiple metabolic substrates to support energy metabolism and cardiac contraction, and exhibit high metabolic flexibility to adapt substrate utilization to distinct developmental and physiological conditions [77]. Fetal and neonatal cardiomyocytes prefer glycolysis, a metabolic state permissive to cell proliferation. Postnatal oxygen elevation drives a metabolic shift toward mitochondrial oxidative phosphorylation and fatty acid oxidation, which coincides with cardiomyocyte maturation, cell cycle exit, and loss of regenerative capacity [15,25]. Developmental metabolic reprogramming is therefore a key determinant of cardiomyocyte proliferative potential [26].

α-KG exerts multifaceted effects that support cardiomyocyte proliferative potential, with epigenetic regulation [59,78,79], antioxidant activity [80], and immune microenvironment homeostasis as its core functional axes [81,82]. We begin with its well-characterized epigenetic regulatory machinery, which forms the primary driver of sustained cell cycle activity in immature cardiomyocytes. As a core tricarboxylic acid cycle intermediate, α-KG acts as an essential cofactor for histone and DNA demethylases [59,78,83]. Fetal cardiomyocytes depend on glycolysis and adequate intracellular α-KG to maintain proliferative phenotypes. After birth, increased tissue oxygen redirects cardiac metabolism toward oxidative phosphorylation and diminishes endogenous α-KG reserves [26]. This metabolic transition suppresses demethylase activity and induces stable transcriptional silencing of cell cycle-associated genes [59]. Multiple studies verify that α-KG triggers JMJD3-mediated demethylation to clear repressive H3K27me3 marks on promoters of proliferation-related genes [59,78]. Exogenous α-KG treatment extends the proliferative window of immature cardiomyocytes and improves cardiac repair following injury [59,78]. This α-KG/JMJD3 regulatory axis is evolutionarily conserved, as it also modulates cardiomyocyte proliferation during zebrafish cardiac development via H3K27me3 suppression and cell cycle gene activation [79]. Apart from epigenetic modulation, α-KG also carries direct antioxidant properties and reshapes macrophage polarization to maintain anti-inflammatory cardiac microenvironments, which align with its triple functional characteristics outlined above [80,81,82]. Existing animal research confirms maternal exercise activates the offspring SOD3-AMPK-IDH cascade and elevates α-KG concentrations in fetal brown adipose tissue, liver, and skeletal muscle [18,19,24]. Nevertheless, no published work has carried out targeted quantification to measure α-KG abundance specifically within fetal myocardium collected from exercise-exposed dams. We synthesize these published findings to put forward merely a preliminary regulatory framework rather than drawing definite causal conclusions. Research specifically investigating α-KG-mediated metabolic control in cardiomyocytes remains scarce, and further empirical work is necessary to validate whether exercise-induced fetal α-KG upregulation coordinates epigenetic and metabolic reprogramming to preserve the proliferative and regenerative capacity of offspring cardiomyocytes.

Collectively, α-KG supports cardiomyocyte proliferation via epigenetic remodeling, antioxidant defense, and a balanced inflammatory microenvironment. While α-KG acts as a robust metabolic remodeler in immune cells, its metabolic regulatory function in cardiomyocytes remains underexplored. Previous studies have confirmed that maternal exercise increases fetal α-KG abundance in fetal brown adipose, liver, and skeletal muscle through AMPK-IDH signaling. However, direct evidence that maternal exercise regulates α-KG abundance in the fetal heart is currently lacking. But these findings provide a rationale for considering α-KG as a candidate mediator through which maternal exercise may influence offspring cardiomyocyte proliferative potential. Future studies should determine whether maternal exercise alters α-KG abundance, α-KG-dependent epigenetic remodeling, or cell-cycle gene expression in fetal and neonatal cardiomyocytes.

## 5. Apelin and α-KG as Complementary Mediators of Maternal Exercise-Induced Cardiomyocyte Proliferation in Offspring

Fetal and postnatal cardiomyocytes exhibit distinct metabolic states. During embryonic development, relatively immature cardiomyocytes rely substantially on glycolysis, whereas postnatal maturation is accompanied by a progressive increase in mitochondrial oxidative phosphorylation [30,75]. These developmental metabolic transitions are closely associated with the decline in cardiomyocyte proliferative capacity after birth. We hypothesize that maternal exercise may influence offspring cardiomyocyte proliferation through two complementary metabolic and signaling pathways involving apelin and α-KG. Maternal exercise increases apelin signaling in the maternal–fetal system, and apelin may promote a glycolytic and pro-proliferative cellular state through pathways involving AMPK and PKM2 [75]. In parallel, maternal exercise-induced placental factors, including SOD3, activate AMPK- and IDH-dependent metabolism, increase α-KG availability [18], and influence epigenetic programs associated with cardiomyocyte cell-cycle regulation [83]. Thus, apelin may act primarily through metabolic signaling, whereas α-KG may function as a link between cellular metabolism and epigenetic regulation. Based on these observations, we propose that apelin- and α-KG-dependent pathways may serve as complementary mediators through which maternal exercise enhances the proliferative potential of offspring cardiomyocytes. However, whether these pathways act independently, sequentially, or cooperatively, and whether they directly regulate cardiomyocyte proliferation in the offspring, remains to be experimentally determined.

## 6. Conclusions

**Figure 3:**
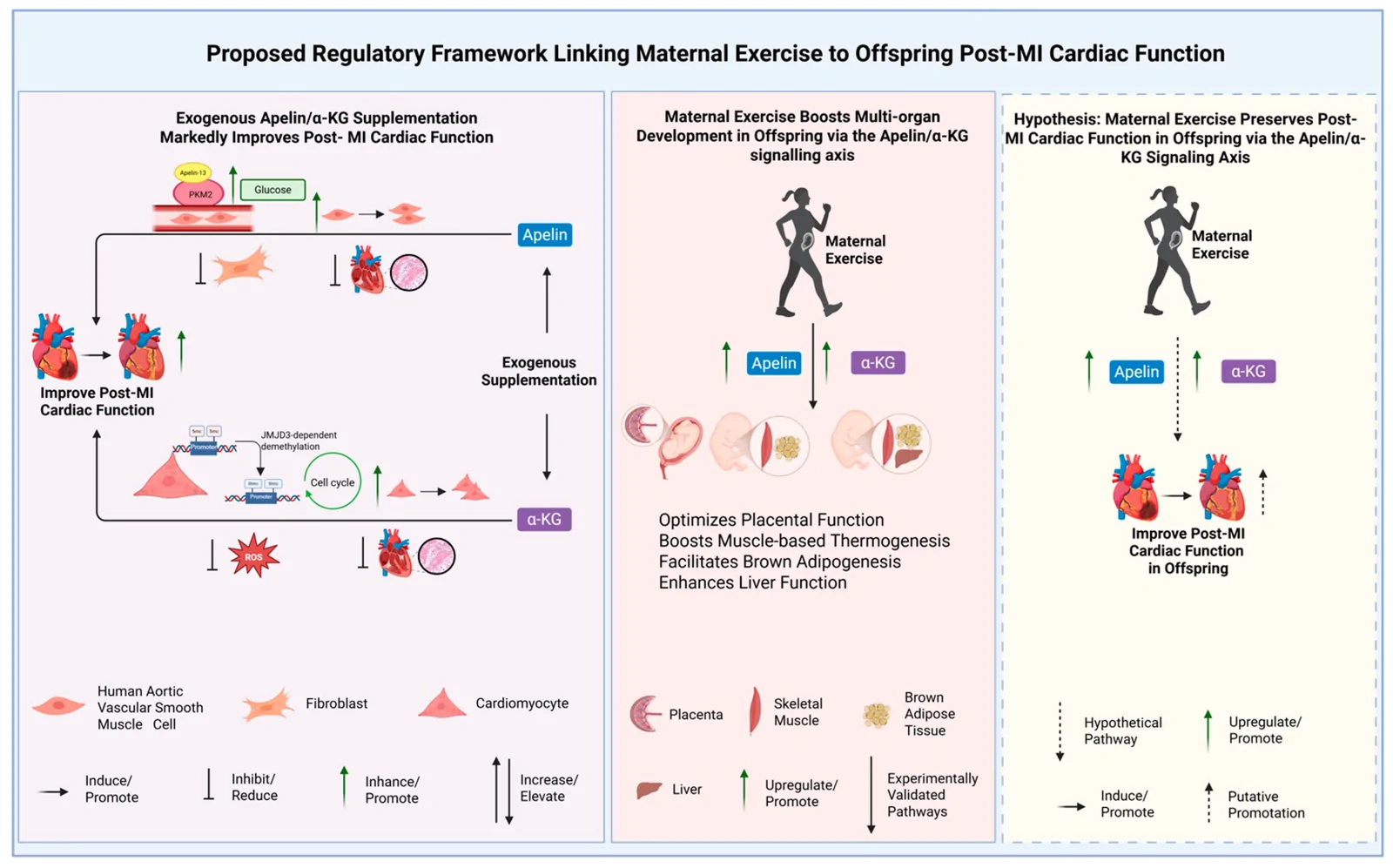
Proposed regulatory framework linking maternal exercise to offspring post-MI cardiac function. The **left** panel illustrates that exogenous apelin/α-KG modulates post- MI cardiac function through multiple mechanisms. The **middle** panel demonstrates that maternal exercise regulates fetal multi-organ development via apelin/α-KG, thereby strengthening metabolic performance and skeletal muscle function in offspring. The **right** panel elaborates on the core hypothesis of this review: maternal exercise may ameliorate post-MI cardiac function in progeny via the apelin/α-KG signaling axis. Solid borders denote experimentally validated outcomes (the **left** and **middle** panels), whereas the dashed border represents the unvalidated hypothetical content (the **right** panel). Created with BioRender.com.

Postnatal cardiomyocyte maturation, metabolic reprogramming, increased ploidy, and sustained repression of cell-cycle genes collectively restrict the regenerative capacity of the adult mammalian heart, creating a major barrier to functional recovery after myocardial injury. The fetal period therefore represents a particularly important developmental window for regulating cardiomyocyte proliferation because cardiomyocytes remain actively proliferative before undergoing postnatal cell-cycle withdrawal. It is important to distinguish fetal/neonatal cardiomyocyte proliferation from final cardiomyocyte number, adult cardiac function, and post-injury cardiac regeneration, as these outcomes are related but not equivalent. Maternal exercise may influence fetal or neonatal cardiomyocyte cell-cycle activity, which could potentially contribute to the final number of cardiomyocytes formed during development. However, a higher cardiomyocyte number does not necessarily guarantee improved baseline cardiac function or enhanced regeneration after injury. Therefore, each step of this proposed model requires independent experimental validation, including assessment of cardiomyocyte proliferation, total cardiomyocyte number, adult cardiac function, and functional recovery after cardiac injury. Maternal exercise is a promising prenatal intervention with broad benefits for offspring health, including improved cognitive function, metabolic homeostasis, exercise capacity, and cardiovascular health. However, whether maternal exercise influences fetal cardiomyocyte proliferation or modifies the long-term regenerative potential of the offspring’s heart remains poorly understood. This review proposes that maternal exercise may regulate offspring cardiomyocyte growth and proliferation through apelin- and α-KG-dependent pathways. Apelin may promote a glycolytic and pro-proliferative cellular state through metabolic signaling, whereas α-KG may link maternal exercise-induced metabolic changes to epigenetic regulation of cardiomyocyte cell-cycle activity (Figure 3). These mechanistic hypotheses remain to be experimentally validated.

## 7. Future Directions

Although limited evidence preliminarily suggests the cardiac protective effects of maternal exercise, direct mechanistic evidence linking prenatal exercise to fetal cardiomyocyte proliferation and long-term cardiac programming remains insufficient. Elucidating these developmental regulatory mechanisms is critical, as prenatal life represents a naturally proliferative window for cardiomyocyte growth. Clarifying how maternal exercise and placental signaling modulate fetal cardiac development may help establish a non-invasive physiological strategy to optimize prenatal cardiomyocyte endowment and improve lifelong cardiac reserve and post-injury regenerative capacity.

Several critical questions remain unaddressed and merit further exploration in future research. Specifically, subsequent studies should precisely distinguish distinct stages of cardiomyocyte cell-cycle progression, including cell-cycle entry, DNA synthesis, mitosis, and cytokinesis, to verify genuine cardiomyocyte expansion rather than merely proliferative activation. Further assessments should characterize final cardiomyocyte abundance, cardiomyocyte maturation status, and potential sex-dependent developmental differences. Long-term follow-up is also essential to determine whether maternal exercise-induced prenatal cardiac adaptations persist into adulthood, enhance basal cardiac function, and improve stress tolerance and myocardial repair capacity after injury. In addition, direct functional validation is required to clarify the independent, sequential, or cooperative roles of apelin and α-KG in mediating fetal cardiac developmental programming. Clarifying these maternal regulatory processes can refine current knowledge regarding early cardiac development and provide tentative biological evidence supporting the beneficial cardiac effects of gestational exercise on offspring long-term cardiac health.

## Data Availability

No new data were created or analyzed in this study. Data sharing is not applicable to this article.

## Abbreviations

The following abbreviations are used in this manuscript:

α-KG	α-Ketoglutarate
AMPK	Adenosine Monophosphate-Activated Protein Kinase
APJ	Angiotensin II Protein J Receptor
CCNA1	Cyclin A1
CCND1	Cyclin D1
CDKs	Cyclin-dependent Kinases
CDK1	Cyclin-dependent Kinase 1
CDK4	Cyclin-dependent Kinase 4
CVD	Cardiovascular Disease
DNA	Deoxyribonucleic Acid
ERBB2	Erb-B2 Receptor Tyrosine Kinase
ERK	Extracellular Signal-Regulated Kinase
FOXM1	Forkhead Box M1
GATA4	GATA Binding Protein 4
GLUT1	Glucose Transporter Type 1
HA-VSMCs	Human Aortic Vascular Smooth Muscle Cells
HIF-1α	Hypoxia-inducible Factor 1α
Hippo-YAP pathway	Hippo-Yes-associated Protein signaling pathway
HOXB13	Homeobox B13
H3K4me3	Histone H3 Lysine 4 Trimethylation
H3K9me3	Histone H3 Lysine 9 Trimethylation
H3K27me3	Histone H3 Lysine 27 Trimethylation
IDH	Isocitrate Dehydrogenase
JMJD3	Jumonji Domain-Containing Protein 3
LDHA	Lactate Dehydrogenase A
MCT1	Monocarboxylate Transporter 1
MCT4	Monocarboxylate Transporter 4
MEIS1	Meis Homeobox 1
MFN2	Mitofusin 2
MI	Myocardial Infarction
mTOR	Mammalian Target of Rapamycin
NRF1	Nuclear Respiratory Factor 1
NRF2	Nuclear Factor Erythroid 2-Related Factor 2
Notch	Notch signaling pathway
PI3K/Akt	Phosphatidylinositol 3-Kinase/Protein Kinase B
PKM2	Pyruvate Kinase M2
P21	Cyclin-dependent Kinase Inhibitor 1A
P27	Cyclin-dependent Kinase Inhibitor 1B
RyR2	Ryanodine Receptor 2
ROS	Reactive Oxygen Species
SOD3	Superoxide Dismutase 3
TBX20	T-box Transcription Factor 20
TCA	Tricarboxylic Acid
Wnt/β-catenin	Wingless-related Integration site/Beta-catenin

## References

[1] Chong B., Jayabaskaran J., Jauhari S.M., Chan S.P., Goh R., Kueh M.T.W., Li H., Chin Y.H., Kong G., Anand V.V. (2025). Global burden of cardiovascular diseases: Projections from 2025 to 2050. Eur. J. Prev. Cardiol..

[2] Velayutham N., Agnew E.J., Yutzey K.E. (2019). Postnatal Cardiac Development and Regenerative Potential in Large Mammals. Pediatr. Cardiol..

[3] Li F., Wang X., Capasso J.M., Gerdes A.M. (1996). Rapid transition of cardiac myocytes from hyperplasia to hypertrophy during postnatal development. J. Mol. Cell. Cardiol..

[4] Mollova M., Bersell K., Walsh S., Savla J., Das L.T., Park S.Y., Silberstein L.E., Dos Remedios C.G., Graham D., Colan S. (2013). Cardiomyocyte proliferation contributes to heart growth in young humans. Proc. Natl. Acad. Sci. USA.

[5] Gunthel M., Barnett P., Christoffels V.M. (2018). Development, Proliferation, and Growth of the Mammalian Heart. Mol. Ther..

[6] Gao Q., Xu L., Cai J. (2021). New drug targets for hypertension: A literature review. Biochim. Biophys. Acta Mol. Basis Dis..

[7] Hao P.P., Jiang F., Chen Y.G., Yang J., Zhang K., Zhang M.X., Zhang C., Zhao Y.X., Zhang Y. (2015). Traditional Chinese medication for cardiovascular disease. Nat. Rev. Cardiol..

[8] Modolo R., Miyazaki Y., Chichareon P., Asano T., Collet C., Tenekecioglu E., Katagiri Y., Soliman O., Garg S., Onuma Y. (2018). Interventional cardiology: Review of the year 2017. EuroIntervention.

[9] Puri R., Chamandi C., Rodriguez-Gabella T., Rodes-Cabau J. (2018). Future of transcatheter aortic valve implantation-evolving clinical indications. Nat. Rev. Cardiol..

[10] Kim Y., Landstrom A.P., Shah S.H., Wu J.C., Seidman C.E., American Heart A. (2024). Gene Therapy in Cardiovascular Disease: Recent Advances and Future Directions in Science: A Science Advisory From the American Heart Association. Circulation.

[11] Nappi F. (2024). Non-Coding RNA-Targeted Therapy: A State-of-the-Art Review. Int. J. Mol. Sci..

[12] Rezaei H., Khadempar S., Farahani N., Hosseingholi E.Z., Hayat S.M.G., Sathyapalan T., Sahebkar A.H. (2020). Harnessing CRISPR/Cas9 technology in cardiovascular disease. Trends Cardiovasc. Med..

[13] Narkhede M., Pardeshi A., Bhagat R., Dharme G. (2024). Review on Emerging Therapeutic Strategies for Managing Cardiovascular Disease. Curr. Cardiol. Rev..

[14] Sakabe M., Matsui H., Sakata H., Ando K., Yamagishi T., Nakajima Y. (2005). Understanding heart development and congenital heart defects through developmental biology: A segmental approach. Congenit. Anom..

[15] Guo Y., Pu W.T. (2020). Cardiomyocyte Maturation: New Phase in Development. Circ. Res..

[16] Gu Y., Zhou Y., Ju S., Liu X., Zhang Z., Guo J., Gao J., Zang J., Sun H., Chen Q. (2022). Multi-omics profiling visualizes dynamics of cardiac development and functions. Cell Rep..

[17] Robinson A.M., Bucci D.J. (2012). Maternal Exercise and Cognitive Functions of the Offspring. Cogn. Sci..

[18] Son J.S., Zhao L., Chen Y., Chen K., Chae S.A., de Avila J.M., Wang H., Zhu M.J., Jiang Z., Du M. (2020). Maternal exercise via exerkine apelin enhances brown adipogenesis and prevents metabolic dysfunction in offspring mice. Sci. Adv..

[19] Kusuyama J., Alves-Wagner A.B., Conlin R.H., Makarewicz N.S., Albertson B.G., Prince N.B., Kobayashi S., Kozuka C., Moller M., Bjerre M. (2021). Placental superoxide dismutase 3 mediates benefits of maternal exercise on offspring health. Cell Metab..

[20] Mohammadkhani R., Khaledi N., Rajabi H., Salehi I., Komaki A. (2020). Influence of the maternal high-intensity-interval-training on the cardiac Sirt6 and lipid profile of the adult male offspring in rats. PLoS ONE.

[21] Xin M., Olson E.N., Bassel-Duby R. (2013). Mending broken hearts: Cardiac development as a basis for adult heart regeneration and repair. Nat. Rev. Mol. Cell Biol..

[22] Karim Rony R.M.I., Tompkins J.D. (2025). Cardiac repair and regeneration: Cell therapy, in vivo reprogramming, and the promise of extracellular vesicles. Exp. Mol. Med..

[23] Son J.S., Chae S.A., Zhao L., Wang H., de Avila J.M., Zhu M.J., Jiang Z., Du M. (2022). Maternal exercise intergenerationally drives muscle-based thermogenesis via activation of apelin-AMPK signaling. EBioMedicine.

[24] Son J.S., Chae S.A., Wang H., Chen Y., Bravo Iniguez A., de Avila J.M., Jiang Z., Zhu M.J., Du M. (2020). Maternal Inactivity Programs Skeletal Muscle Dysfunction in Offspring Mice by Attenuating Apelin Signaling and Mitochondrial Biogenesis. Cell Rep..

[25] Magadum A., Singh N., Kurian A.A., Munir I., Mehmood T., Brown K., Sharkar M.T.K., Chepurko E., Sassi Y., Oh J.G. (2020). Pkm2 Regulates Cardiomyocyte Cell Cycle and Promotes Cardiac Regeneration. Circulation.

[26] Lopaschuk G.D., Jaswal J.S. (2010). Energy metabolic phenotype of the cardiomyocyte during development, differentiation, and postnatal maturation. J. Cardiovasc. Pharmacol..

[27] Porrello E.R., Mahmoud A.I., Simpson E., Hill J.A., Richardson J.A., Olson E.N., Sadek H.A. (2011). Transient regenerative potential of the neonatal mouse heart. Science.

[28] Ye L., D’Agostino G., Loo S.J., Wang C.X., Su L.P., Tan S.H., Tee G.Z., Pua C.J., Pena E.M., Cheng R.B. (2018). Early Regenerative Capacity in the Porcine Heart. Circulation.

[29] Bae J., Salamon R.J., Brandt E.B., Paltzer W.G., Zhang Z., Britt E.C., Hacker T.A., Fan J., Mahmoud A.I. (2021). Malonate Promotes Adult Cardiomyocyte Proliferation and Heart Regeneration. Circulation.

[30] Oka T., Lam V.H., Zhang L., Keung W., Cadete V.J., Samokhvalov V., Tanner B.A., Beker D.L., Ussher J.R., Huqi A. (2012). Cardiac hypertrophy in the newborn delays the maturation of fatty acid beta-oxidation and compromises postischemic functional recovery. Am. J. Physiol. Heart Circ. Physiol..

[31] Puente B.N., Kimura W., Muralidhar S.A., Moon J., Amatruda J.F., Phelps K.L., Grinsfelder D., Rothermel B.A., Chen R., Garcia J.A. (2014). The oxygen-rich postnatal environment induces cardiomyocyte cell-cycle arrest through DNA damage response. Cell.

[32] Garbern J.C., Lee R.T. (2021). Mitochondria and metabolic transitions in cardiomyocytes: Lessons from development for stem cell-derived cardiomyocytes. Stem Cell Res. Ther..

[33] Anatskaya O.V., Vinogradov A.E. (2022). Polyploidy as a Fundamental Phenomenon in Evolution, Development, Adaptation and Diseases. Int. J. Mol. Sci..

[34] Abouleisa R.R.E., Salama A.B.M., Ou Q., Tang X.L., Solanki M., Guo Y., Nong Y., McNally L., Lorkiewicz P.K., Kassem K.M. (2022). Transient Cell Cycle Induction in Cardiomyocytes to Treat Subacute Ischemic Heart Failure. Circulation.

[35] Kikuchi K., Holdway J.E., Werdich A.A., Anderson R.M., Fang Y., Egnaczyk G.F., Evans T., Macrae C.A., Stainier D.Y., Poss K.D. (2010). Primary contribution to zebrafish heart regeneration by gata4(+) cardiomyocytes. Nature.

[36] Jopling C., Sleep E., Raya M., Marti M., Raya A., Izpisua Belmonte J.C. (2010). Zebrafish heart regeneration occurs by cardiomyocyte dedifferentiation and proliferation. Nature.

[37] Monroe T.O., Hill M.C., Morikawa Y., Leach J.P., Heallen T., Cao S., Krijger P.H.L., de Laat W., Wehrens X.H.T., Rodney G.G. (2019). YAP Partially Reprograms Chromatin Accessibility to Directly Induce Adult Cardiogenesis In Vivo. Dev. Cell.

[38] Laflamme M.A., Murry C.E. (2011). Heart regeneration. Nature.

[39] Kimura W., Xiao F., Canseco D.C., Muralidhar S., Thet S., Zhang H.M., Abderrahman Y., Chen R., Garcia J.A., Shelton J.M. (2015). Hypoxia fate mapping identifies cycling cardiomyocytes in the adult heart. Nature.

[40] Di Stefano V., Giacca M., Capogrossi M.C., Crescenzi M., Martelli F. (2011). Knockdown of cyclin-dependent kinase inhibitors induces cardiomyocyte re-entry in the cell cycle. J. Biol. Chem..

[41] Rojas A., Kong S.W., Agarwal P., Gilliss B., Pu W.T., Black B.L. (2008). GATA4 is a direct transcriptional activator of cyclin D2 and Cdk4 and is required for cardiomyocyte proliferation in anterior heart field-derived myocardium. Mol. Cell. Biol..

[42] Malek Mohammadi M., Kattih B., Grund A., Froese N., Korf-Klingebiel M., Gigina A., Schrameck U., Rudat C., Liang Q., Kispert A. (2017). The transcription factor GATA4 promotes myocardial regeneration in neonatal mice. EMBO Mol. Med..

[43] Boogerd C.J., Zhu X., Aneas I., Sakabe N., Zhang L., Sobreira D.R., Montefiori L., Bogomolovas J., Joslin A.C., Zhou B. (2018). Tbx20 Is Required in Mid-Gestation Cardiomyocytes and Plays a Central Role in Atrial Development. Circ. Res..

[44] Fang Y., Lai K.S., She P., Sun J., Tao W., Zhong T.P. (2020). Tbx20 Induction Promotes Zebrafish Heart Regeneration by Inducing Cardiomyocyte Dedifferentiation and Endocardial Expansion. Front. Cell Dev. Biol..

[45] Xiang F.L., Guo M., Yutzey K.E. (2016). Overexpression of Tbx20 in Adult Cardiomyocytes Promotes Proliferation and Improves Cardiac Function After Myocardial Infarction. Circulation.

[46] Zuppo D.A., Missinato M.A., Santana-Santos L., Li G., Benos P.V., Tsang M. (2023). Foxm1 regulates cardiomyocyte proliferation in adult zebrafish after cardiac injury. Development.

[47] Mahmoud A.I., Kocabas F., Muralidhar S.A., Kimura W., Koura A.S., Thet S., Porrello E.R., Sadek H.A. (2013). Meis1 regulates postnatal cardiomyocyte cell cycle arrest. Nature.

[48] Nguyen N.U.N., Canseco D.C., Xiao F., Nakada Y., Li S., Lam N.T., Muralidhar S.A., Savla J.J., Hill J.A., Le V. (2020). A calcineurin-Hoxb13 axis regulates growth mode of mammalian cardiomyocytes. Nature.

[49] Liu S., Li K., Wagner Florencio L., Tang L., Heallen T.R., Leach J.P., Wang Y., Grisanti F., Willerson J.T., Perin E.C. (2021). Gene therapy knockdown of Hippo signaling induces cardiomyocyte renewal in pigs after myocardial infarction. Sci. Transl. Med..

[50] Garbern J.C., Helman A., Sereda R., Sarikhani M., Ahmed A., Escalante G.O., Ogurlu R., Kim S.L., Zimmerman J.F., Cho A. (2020). Inhibition of mTOR Signaling Enhances Maturation of Cardiomyocytes Derived From Human-Induced Pluripotent Stem Cells via p53-Induced Quiescence. Circulation.

[51] Humphrey J.D., Dufresne E.R., Schwartz M.A. (2014). Mechanotransduction and extracellular matrix homeostasis. Nat. Rev. Mol. Cell Biol..

[52] Collesi C., Zentilin L., Sinagra G., Giacca M. (2008). Notch1 signaling stimulates proliferation of immature cardiomyocytes. J. Cell Biol..

[53] Collesi C., Felician G., Secco I., Gutierrez M.I., Martelletti E., Ali H., Zentilin L., Myers M.P., Giacca M. (2018). Reversible Notch1 acetylation tunes proliferative signalling in cardiomyocytes. Cardiovasc. Res..

[54] Ye S., Wang C., Xu Z., Lin H., Wan X., Yu Y., Adhicary S., Zhang J.Z., Zhou Y., Liu C. (2023). Impaired Human Cardiac Cell Development due to NOTCH1 Deficiency. Circ. Res..

[55] Heallen T., Zhang M., Wang J., Bonilla-Claudio M., Klysik E., Johnson R.L., Martin J.F. (2011). Hippo pathway inhibits Wnt signaling to restrain cardiomyocyte proliferation and heart size. Science.

[56] D’Uva G., Aharonov A., Lauriola M., Kain D., Yahalom-Ronen Y., Carvalho S., Weisinger K., Bassat E., Rajchman D., Yifa O. (2015). ERBB2 triggers mammalian heart regeneration by promoting cardiomyocyte dedifferentiation and proliferation. Nat. Cell Biol..

[57] Wang J., Liu S., Heallen T., Martin J.F. (2018). The Hippo pathway in the heart: Pivotal roles in development, disease, and regeneration. Nat. Rev. Cardiol..

[58] Fajardo V.M., Feng I., Chen B.Y., Perez-Ramirez C.A., Shi B., Clark P., Tian R., Lien C.L., Pellegrini M., Christofk H. (2021). GLUT1 overexpression enhances glucose metabolism and promotes neonatal heart regeneration. Sci. Rep..

[59] Li X., Wu F., Gunther S., Looso M., Kuenne C., Zhang T., Wiesnet M., Klatt S., Zukunft S., Fleming I. (2023). Publisher Correction: Inhibition of fatty acid oxidation enables heart regeneration in adult mice. Nature.

[60] Segabinazi E., Spindler C., Meireles A.L.F., Piazza F.V., Mega F., Salvalaggio G.D.S., Achaval M., Marcuzzo S. (2019). Effects of Maternal Physical Exercise on Global DNA Methylation and Hippocampal Plasticity of Rat Male Offspring. Neuroscience.

[61] Shi H., Li J., Li F., Yu H., Zhang F., Wu T., Yang L., Li Y., Hu R., Chen M. (2025). Vitamin C-Dependent Intergenerational Inheritance of Enhanced Endurance Performance Following Maternal Exercise. Adv. Sci..

[62] Pinckard K.M., Felix-Soriano E., Hamilton S., Terentyeva R., Baer L.A., Wright K.R., Nassal D., Esteves J.V., Abay E., Shettigar V.K. (2024). Maternal exercise preserves offspring cardiovascular health via oxidative regulation of the ryanodine receptor. Mol. Metab..

[63] Beeson J.H., Blackmore H.L., Carr S.K., Dearden L., Duque-Guimaraes D.E., Kusinski L.C., Pantaleao L.C., Pinnock A.G., Aiken C.E., Giussani D.A. (2018). Maternal exercise intervention in obese pregnancy improves the cardiovascular health of the adult male offspring. Mol. Metab..

[64] Chung E., Joiner H.E., Skelton T., Looten K.D., Manczak M., Reddy P.H. (2017). Maternal exercise upregulates mitochondrial gene expression and increases enzyme activity of fetal mouse hearts. Physiol. Rep..

[65] Saiyin T., Engineer A., Greco E.R., Kim M.Y., Lu X., Jones D.L., Feng Q. (2019). Maternal voluntary exercise mitigates oxidative stress and incidence of congenital heart defects in pre-gestational diabetes. J. Cell. Mol. Med..

[66] May L.E., Glaros A., Yeh H.W., Clapp J.F., Gustafson K.M. (2010). Aerobic exercise during pregnancy influences fetal cardiac autonomic control of heart rate and heart rate variability. Early Hum. Dev..

[67] May L.E., McDonald S., Forbes L., Jones R., Newton E., Strickland D., Isler C., Haven K., Steed D., Kelley G. (2020). Influence of maternal aerobic exercise during pregnancy on fetal cardiac function and outflow. Am. J. Obstet. Gynecol. MFM.

[68] May L.E., Scholtz S.A., Suminski R., Gustafson K.M. (2014). Aerobic exercise during pregnancy influences infant heart rate variability at one month of age. Early Hum. Dev..

[69] Salvesen K.A., Hem E., Sundgot-Borgen J. (2012). Fetal wellbeing may be compromised during strenuous exercise among pregnant elite athletes. Br. J. Sports Med..

[70] Moolyk A.N., Wilson M.K., Matenchuk B.A., Bains G., Gervais M.J., Wowdzia J.B., Davenport M.H. (2025). Maternal and fetal responses to acute high-intensity resistance exercise during pregnancy. Br. J. Sports Med..

[71] Zheng J., Alves-Wagner A.B., Stanford K.I., Prince N.B., So K., Mul J.D., Dirice E., Hirshman M.F., Kulkarni R.N., Goodyear L.J. (2020). Maternal and paternal exercise regulate offspring metabolic health and beta cell phenotype. BMJ Open Diabetes Res. Care.

[72] Tang L., Qiu H., Xu B., Su Y., Nyarige V., Li P., Chen H., Killham B., Liao J., Adam H. (2024). Microparticle Mediated Delivery of Apelin Improves Heart Function in Post Myocardial Infarction Mice. Circ. Res..

[73] Chandrasekaran B., Dar O., McDonagh T. (2008). The role of apelin in cardiovascular function and heart failure. Eur. J. Heart Fail..

[74] Wang W., McKinnie S.M., Patel V.B., Haddad G., Wang Z., Zhabyeyev P., Das S.K., Basu R., McLean B., Kandalam V. (2013). Loss of Apelin exacerbates myocardial infarction adverse remodeling and ischemia-reperfusion injury: Therapeutic potential of synthetic Apelin analogues. J. Am. Heart Assoc..

[75] Geiger K., Muendlein A., Stark N., Saely C.H., Wabitsch M., Fraunberger P., Drexel H. (2011). Hypoxia induces apelin expression in human adipocytes. Horm. Metab. Res..

[76] Zhou Q., Xu J., Liu M., He L., Zhang K., Yang Y., Yang X., Zhou H., Tang M., Lu L. (2019). Warburg effect is involved in apelin-13-induced human aortic vascular smooth muscle cells proliferation. J. Cell. Physiol..

[77] Kolwicz S.C., Purohit S., Tian R. (2013). Cardiac metabolism and its interactions with contraction, growth, and survival of cardiomyocytes. Circ. Res..

[78] Shi Y., Tian M., Zhao X., Tang L., Wang F., Wu H., Liao Q., Ren H., Fu W., Zheng S. (2024). α-Ketoglutarate promotes cardiomyocyte proliferation and heart regeneration after myocardial infarction. Nat. Cardiovasc. Res..

[79] Akerberg A.A., Henner A., Stewart S., Stankunas K. (2017). Histone demethylases Kdm6ba and Kdm6bb redundantly promote cardiomyocyte proliferation during zebrafish heart ventricle maturation. Dev. Biol..

[80] Liu S., He L., Yao K. (2018). The Antioxidative Function of Alpha-Ketoglutarate and Its Applications. Biomed. Res. Int..

[81] Zhou B., Magana L., Hong Z., Huang L.S., Chakraborty S., Tsukasaki Y., Huang C., Wang L., Di A., Ganesh B. (2020). The angiocrine Rspondin3 instructs interstitial macrophage transition via metabolic-epigenetic reprogramming and resolves inflammatory injury. Nat. Immunol..

[82] Ming-Chin Lee K., Achuthan A.A., De Souza D.P., Lupancu T.J., Binger K.J., Lee M.K.S., Xu Y., McConville M.J., de Weerd N.A., Dragoljevic D. (2022). Type I interferon antagonism of the JMJD3-IRF4 pathway modulates macrophage activation and polarization. Cell Rep..

[83] Lalowski M.M., Bjork S., Finckenberg P., Soliymani R., Tarkia M., Calza G., Blokhina D., Tulokas S., Kankainen M., Lakkisto P. (2018). Characterizing the Key Metabolic Pathways of the Neonatal Mouse Heart Using a Quantitative Combinatorial Omics Approach. Front. Physiol..

